# An investigation of alkaline phosphatase enzymatic activity after electrospinning and electrospraying

**DOI:** 10.1016/j.jddst.2021.102592

**Published:** 2021-08

**Authors:** Lesley C. Onyekuru, Anabela Moreira, Jiazhe Zhang, Ukrit Angkawinitwong, Pedro F. Costa, Steve Brocchini, Gareth R. Williams

**Affiliations:** a UCL School of Pharmacy, University College London, 29-39 Brunswick Square, London, WC1N 1AX, UK; b Biofabics Lda., Rua Alfredo Allen 455, 4200-135, Porto, Portugal; c UCL Institute of Ophthalmology, University College London, 11-43 Bath Street, London, EC1V 9EL, UK

**Keywords:** Electrohydrodynamic processing, Electrospinning, Electrospraying, Alkaline phosphatase, Protein delivery systems

## Abstract

The high target specificity and multifunctionality of proteins has led to great interest in their clinical use. To this end, the development of delivery systems capable of preserving their bioactivity and improving bioavailability is pivotal to achieve high effectiveness and satisfactory therapeutic outcomes. Electrohydrodynamic (EHD) techniques, namely electrospinning and electrospraying, have been widely explored for protein encapsulation and delivery. In this work, monoaxial and coaxial electrospinning and electrospraying were used to encapsulate alkaline phosphatase (ALP) into poly(ethylene oxide) fibres and particles, respectively, and the effects of the processing techniques on the integrity and bioactivity of the enzyme were assessed. A full morphological and physicochemical characterisation of the blend and core-shell products was performed. ALP was successfully encapsulated within monolithic and core-shell electrospun fibres and electrosprayed particles, with drug loadings and encapsulation efficiencies of up to 21% and 99%, respectively. Monoaxial and coaxial electrospinning were equally effective in preserving ALP function, leading to no activity loss compared to fresh aqueous solutions of the enzyme. While the same result was observed for monoaxial electrospraying, coaxial electrospraying of ALP caused a 40% reduction in its bioactivity, which was attributed to the high voltage (22.5 kV) used during processing. This demonstrates that choosing between blend and coaxial EHD processing for protein encapsulation is not always straightforward, being highly dependent on the chosen therapeutic agent and the effects of the processing conditions on its bioactivity.

## Highlights

• Blend and coaxial electrohydrodynamic techniques can be used for alkaline phosphatase (ALP) encapsulation.
• Both blend and coaxial electrospinning equally preserve ALP bioactivity at ca. 100%.
• ALP activity decreased after coaxial electrospraying, but not blend electrospraying.
• The high voltages needed for coaxial electrospraying appear to affect ALP bioactivity.

## 1 Introduction

Interest in therapy using protein-based active ingredients has been rising steadily over the years. Unlike most small molecule drugs, peptides and proteins act with high specificity towards their target, potentially decreasing adverse and systemic side effects [1]. Further, advances in computational modelling and proteomic research increasingly allow for recombinant protein development and predictions of protein stability and biological activity [2]. However, the administration of protein-based therapeutic agents – biologics – is still associated with a number of limitations. Biologics are typically unstable in the gut, poorly absorbed orally, and can easily be degraded into constituent peptides or amino acids, reducing their bioavailability. As such, their administration is usually performed parenterally. Since proteins have short half-lives in the circulation, repeated administrations are required to maintain adequate doses at the target site. This unstable nature of proteins, as well as their low ability to cross biological barriers, further hinders their therapeutic effect [3]. Therefore, the generation of protein delivery systems capable of improving bioavailability, protecting their effector or catalytic activity, and maintaining therapeutic doses over extended periods of time is required.

**Fig. 1:**
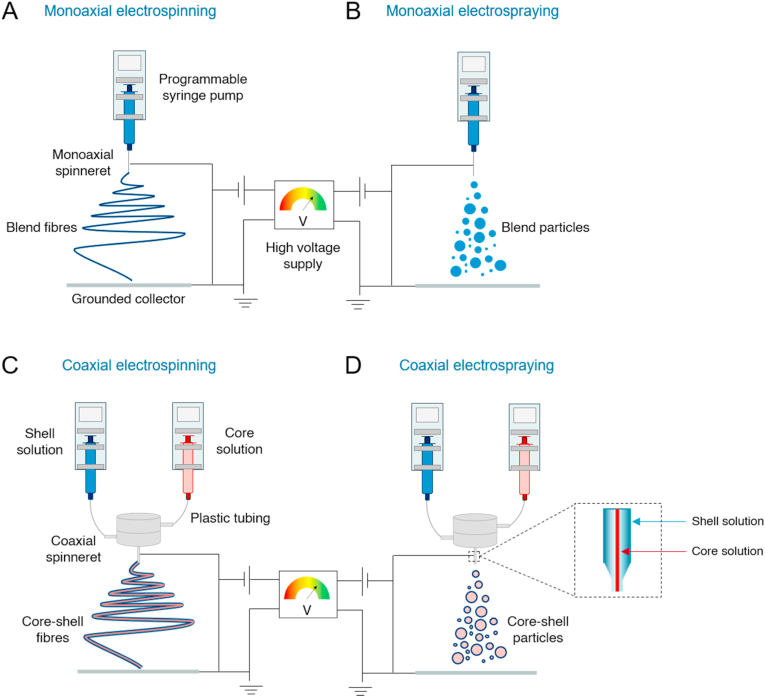
Monoaxial (A, B) and coaxial (C, D) electrospinning and electrospraying. These methods are based on the extrusion of a polymer solution or melt through a spinneret, to which is applied a high potential difference, causing the ejection of solution towards a grounded metal collector. During this trajectory, solvent evaporates and dry fibres (A, C) or particles (B, D) are deposited, depending on solution and processing conditions. Monoaxial electrospinning and spraying give rise to monolithic fibres and particles, respectively, while coaxial EHD processing generates core-shell structures.

Electrohydrodynamic (EHD) processing is based on the extrusion of a polymer solution, emulsion, or melt under a strong electric field for the production of fibres – electrospinning – or particles – electrospraying (Fig. 1). Both of these techniques have been extensively explored for drug delivery: though electrospinning has been more widely studied than electrospraying, both can be tailored to provide formulations with structural and functional advantages over the active pharmaceutical ingredient alone [4]. EHD processing is a one-step methodology that allows the production of drug delivery systems with high surface area-to-volume ratios and nano- or microscaled dimensions [5]. In part as a result of the greater volume of work performed, electrospinning processes are currently more practical and easier to optimise than electrospraying [6,7]. Large-scale manufacturing is also at present easier with electrospinning [8]. The processes for making particles and fibres are largely dependent on the polymer solution viscosity, and the same polymer and drug materials can be processed both by electrospinning and electrospraying. Compared to electrospun fibres, the preparation of electrosprayed particles requires lower solution viscosity. This is typically achieved by reducing the polymer molecular weight and/or concentration [9,10].

Blend or monoaxial EHD processes (Fig. 1A and B) simply disperse the bioactive components directly into the polymer working fluid [11], resulting in monolithic particles or fibres. With these techniques, there is minimal control over protein distribution within the resultant products [12]. However, blend EHD methods are less complex than coaxial processes (Fig. 1C and D), in which a specialised two-needle spinneret is required to generate core-shell particle and fibre architectures. With this core-shell organisation, it is possible to confine protein localisation to the core to minimise burst release, so the system can act as a reservoir [[13], [14], [15]]. For instance, lactate dehydrogenase has been loaded into poly(vinyl alcohol) (PVA) fibres using coaxial electrospinning, and sustained delivery over 20 days was observed [16]. Additionally, biomolecules encapsulated within the core may be better protected from degradation and have minimal interaction with organic solvents during fabrication [17,18]. This is in contrast to using a single solution for EHD processing, where the protein and solvent inevitably come into contact. Protein exposure to organic solvents generally causes the loss of tertiary structure, resulting in denaturation and loss of function [19]. For instance, Mickova et al. encapsulated horseradish peroxidase-containing liposomes in electrospun fibres [20]. The nanofibre-liposome systems were either blended with PVA or encapsulated as core-shell materials, with PVA and liposomes forming the core and poly-ε-caprolactone (PCL) as the shell. They found that liposomes embedded within core-shell fibres better preserved the enzymatic activity of horseradish peroxidase, while the blend system did not preserve the intact liposomes and resulted in a loss of enzymatic activity [20].

EHD processing is also advantageous in terms of the broad range of natural and synthetic biomaterials that can be used for fibre and particle formation [4]. Poly(ethylene oxide) (PEO) is a synthetic [21] semi-crystalline, non-toxic, and biocompatible [22,23] polymer used in cosmetics [24], food additives, biomaterials, and drug formulation [25]. The physicochemical properties of the polymer, such as its viscosity in solution, solubility, and the wide range of molecular weights available, make it suitable for both electrospinning and electrospraying [26]. PEO has been used extensively for EHD fabrication of protein-loaded biomaterials [27,28].

The suitability of EHD processing for protein encapsulation has been proven with many biologics (recently reviewed in refs. [29,30]), including bovine serum albumin [31], growth factors [[32], [33], [34]], hormones [35], and enzymes such as lysozyme [36] and alkaline phosphatase (ALP) [12]. ALP is a dimeric metalloenzyme with a molecular weight of about 115–165 kDa [37,38], where two identical subunits of about 56 kDa act to catalyse the hydrolysis of phosphate monoester and diester bonds in alkaline environments [39]. ALP is found in the placenta, liver, intestinal mucosa, kidneys, neuronal membranes, bones, tumoral tissue and even in bacterial species such as *Escherichia coli* [[40], [41], [42]]. In the intestine, ALP performs a protective, anti-inflammatory action by detoxifying lipopolysaccharide (LPS, a component of the outer membrane of Gram-negative bacteria), adjusting the duodenal pH, and contributing to the regulation of the gut microbiota, suggesting a potential therapeutic application of the enzyme in intestinal inflammatory and infectious disorders [43,44]. Earlier studies indicate that ALP's ability for LPS detoxification could also give it a role in the treatment of sepsis [45,46].

A study by Ji et al. has previously explored ALP encapsulation in blend and coaxial electrospinning in PCL and poly(ethylene glycol) fibres [12]. The fibres fabricated as core-shell formulations retained about 76% of the enzymatic activity, while the blend fibres displayed only 49% of the fresh ALP activity. The core-shell fibres also displayed uniform morphology with a lower burst release than the blend fibres. Here, ALP will be used as a model to understand the effects of EHD processing on the activity of an encapsulated protein, since it is an extensively researched and widely available enzyme with a fairly simple activity assay [12,47,48]. Due to its high aqueous solubility, post-processing recovery of the enzyme from a PEO-based delivery system should be straightforward, therefore facilitating the assessment of protein activity after the encapsulation procedure. Hence, the principal aim of this work was to examine the potential of using EHD processing techniques to fabricate protein-loaded particles and fibres using ALP as a model drug. The specific objectives were to optimise the formation of ALP-loaded fibres and particles using both monoaxial and coaxial EHD processing methods, examine the impacts of the techniques on the physical properties of the fibres and particles, and explore how the different processing approaches impact on ALP activity.

## 2 Methods

The formulations prepared in this study and their nomenclature are detailed in Table 1.

**Table 1:** Data key for the ALP-loaded fibres and particles described in this paper.

Sample details	Key used
Fibres prepared using coaxial electrospinning	EFC
Fibres prepared using blend electrospinning	EFB
Particles prepared using coaxial electrospraying	EPC
Particles prepared using blend electrospraying	EPB
Alkaline phosphatase, as supplied	ALP

### 2.1 Optimisation of electrospinning parameters

The protocol followed to optimise the electrospinning parameters was adapted from Jin's study [49]. PEO (600 kDa, Sigma-Aldrich, UK) solutions (3% w/v in ethanol:water, 7:3 v/v) were prepared and stirred for 48 h at 30 °C to obtain homogeneous mixtures. PEO/enzyme solutions were generated by adding ALP (Sigma-Aldrich, UK) solution (5% w/v in 0.5 mL phosphate buffered saline (PBS, pH 7.4, Thermo Fisher Scientific, UK)) to 3.5 mL of PEO solution and gently stirring until homogeneous, immediately prior to spinning. For monoaxial blend electrospinning (EFB fibres), PEO or PEO/enzyme solutions were loaded in plastic syringes and spun from a 0.61 mm inner diameter needle (20G) using a voltage range of 9–15 kV, a needle-to-collector plate distance range of 12–22.5 cm, and a flow rate of 0.5–1 mL/h. Fibres were collected as a mat on aluminium foil, left for 2 h at room temperature to allow for additional solvent evaporation, and then stored in a desiccator over phosphorous pentoxide prior to further analysis and characterisation.

Coaxial (EFC) fibres were spun with 3% w/v PEO (shell solution, ethanol:water, 7:3 v/v) and 5% w/v ALP (core solution, PBS), using a coaxial spinneret with inner diameters of 2 mm (outer capillary) and 1 mm (inner capillary). The core flow rate was varied from 0.1 to 0.5 mL/h, and the shell flow rate from 0.6 to 1.0 mL/h. The voltage range explored was 9–18 kV and the distance 12–20 cm. The resulting fibres were collected and stored in the same way blend fibres were. The conditions for monoaxial and coaxial spinning are described in Table 2 and Table 3, respectively.

**Table 2:** Optimised processing conditions for the formation of EFB.

Process parameter	Value
Voltage	10 kV
Distance	22.5 cm
Flow rate	0.8 mL/h

**Table 3:** Optimised processing conditions for the formation of EFC.

Process parameter	Value
Voltage	12 kV
Distance	15 cm
Flow rate (core)	0.1 mL/h
Flow rate (shell)	0.6 mL/h

### 2.2 Optimisation of electrospraying parameters

For blend electrospraying (EPB particles), 250 μL of ALP (10% w/v in PBS) was added to a PEO (20 kDa, Sigma-Aldrich, UK) solution (10% w/v in ethanol:water, 7:3 v/v, 3.75 mL) to form the spraying fluid, which was then loaded into a disposable plastic syringe. The latter was infused with a flow rate range of 0.3–1.5 mL/h, a needle-to-collector plate distance of 10–20 cm and an applied voltage from 9 to 25 kV. For coaxial electrospraying (EPC), the shell fluid was a PEO solution at 10% w/v in ethanol:water (7:3 v/v), and ALP (5% w/v) was dissolved in PBS and used as the core solution. The ALP and polymer solutions were freshly prepared before electrospraying. ALP/PEO core-shell particles were prepared using a coaxial needle (core internal diameter 1 mm, and shell internal diameter 2 mm), through which the two fluids were simultaneously dispensed. The shell flow rate was varied from 0.6 to 1.0 mL/h, and the core flow rate from 0.02 to 0.15 mL/h. The voltage range explored was 17–23 kV and the distance 15–20 cm. The conditions for blend and coaxial spraying are described in Table 4 and Table 5, respectively.

**Table 4:** Optimised processing conditions for the formation of EPB.

Process parameter	Value
Voltage	15.5 kV
Distance	15 cm
Flow rate	0.6 mL/h

**Table 5:** Optimised processing conditions for the formation of EPC.

Process parameter	Value
Voltage	22.5 kV
Distance	17 cm
Flow rate (core)	0.02 mL/h
Flow rate (shell)	0.3 mL/h

Monoaxial and coaxial electrospinning and electrospraying were carried at room temperature (25 ± 2 °C) and at a relative humidity of 30 ± 1%. At least three batches of each formulation were prepared to ensure reproducibility.

### 2.3 Morphological characterisation

#### 2.3.1 Scanning electron microscopy (SEM)

For electrospun fibres, small samples of approximately 1 × 1 cm were cut from each mat. For particles, a similar area of aluminium foil with deposited particles was used. Samples were then coated with a 20 nm gold sputter (Q150T, Quorum Technologies, UK) and imaged using a Quanta 200F microscope (FEI, USA) connected to a secondary electron detector (Everheart-Thornley detector). Following imaging, the ImageJ software (National Institutes of Health, USA) was used to determine the average fibre or particle diameter [50]. At least 100 separate measurements for each sample were obtained. The collected data were then plotted into a histogram using OriginLab software (Version 9.1, Origin OEM, USA).

#### 2.3.2 Transmission electron microscopy (TEM)

During the particle fabrication process, samples were collected directly onto formvar coated 2030C 300 mesh copper TEM grids (SPI Supplies, USA) for about 20 s. The samples were then analysed using a JEM-2100F instrument (JEOL, Japan).

### 2.4 Physicochemical characterisation

#### 2.4.1 Fourier transform infra-red spectroscopy (FTIR)

FTIR was performed in attenuated total reflectance (ATR) mode on approximately 3 mg of samples using a Spectrum 100 spectrometer (Perkin Elmer, USA), with twenty scans collected per sample at a resolution of 2 cm^−1^ over the wavelength range of 4000–650 cm^−1^. Three independent samples were investigated per formulation, and one representative spectrum is shown for each.

#### 2.4.2 X-ray powder diffraction (XRD)

Samples were placed on aluminium plates and diffraction patterns were obtained using a Miniflex 600 instrument (Rigaku, Japan) supplied with Cu Kα radiation at 40 kV and 15 mA. Patterns were recorded over the 2θ range 3–40° at a speed of 5° per minute (size step = 0.02°). Three independent samples were investigated per formulation, and one representative pattern is shown for each.

#### 2.4.3 Differential scanning calorimetry (DSC)

Approximately 5 mg of each sample were sealed in Tzero aluminium pans (T130425) with pin-holed hermetic lids (TA instruments, USA). A Q2000 DSC (TA Instruments) was used to collect data at a heating rate of 10 °C per minute, from 20 °C to ca. 140 °C. All experiments were performed under a nitrogen purge of 50 mL/min. Three independent samples were investigated per formulation, and one representative DSC thermogram for each is presented.

### 2.5 Protein quantification and activity assessment

#### 2.5.1 Sodium dodecyl sulphate polyacrylamide gel electrophoresis (SDS-PAGE)

NuPAGE® (6 μL) LDS sample buffer (Invitrogen, Thermo Fisher Scientific, UK) was added to a solution (20 μL) of the protein to be tested. The mixture was vortexed for 3–5 s and loaded onto a Novex® Bis-Tris 4–12% precast gel (Invitrogen, Thermo Fisher Scientific, UK) mounted in an electrophoresis tank. The pre-stained molecular weight standard (5 μL) was added to the first well. Running buffer (NuPAGE® MOPS SDS buffer diluted in distilled water, 1:20 v/v; Novex®, Life Technologies, UK) was added into the tank. A voltage of 200 V and current of 70 mA were applied and the experiment allowed to run for 50 min. The gel was removed, stained using Coomassie blue for 1 h, and then washed with distilled water for 1 h.

#### 2.5.2 MicroBCA™

To quantify the protein present in a sample, the Pierce™ Micro BCA Protein Assay Kit (ThermoFisher Scientific, UK) was used, following the manufacturer's recommended protocol. Assays were performed in clear 96-well plates (Corning, USA). To make the MicroBCA™ working reagent (WR), the provided reagents A, B and C were mixed in the ratio 25:24:1 v/v/v. Protein samples of 150 μL were added to individual wells of the plate, followed by an equal volume of WR. The plate was shaken using a plate shaker for 30 s. Controls included the polymer used in processing, no treatment (PBS), ALP, and the solvents used for processing. After shaking, the plate was incubated at 37 °C for 2 h, left to cool at room temperature for 5 min, and read for absorbance at 562 nm with a SpectraMax M2e plate reader (Molecular Devices, USA). The most accurate quantification range using BCA lies within the concentration range of 5–40 μg/mL. Thus, to quantify the protein present, a standard curve was made for ALP across this range.

#### 2.5.3 Drug loading

The drug loading was calculated using Equation (1), with protein mass determined using the MicroBCA™ method as detailed in Section 2.5.2.

Equation 1

(1) $$DL\%=\frac{mass\;of\;protein\;in\;particles\;or\;fibres}{total\;weight\;of\;sample}\times 100$$

##### 2.5.3.1 Encapsulation efficiency

Encapsulation efficiency was calculated using Equation (2), again based on protein quantifications from the MicroBCA™ assay.

Equation 2

(2) $$EE\%=\frac{total\;mass\;of\;protein\;extracted}{theoretical\;mass\;of\;protein\;in\;the\;formulation}\times 100$$

##### 2.5.3.2 Activity assay

ALP activity was measured using a p-nitrophenyl phosphate (p-NPP) substrate. The colourless p-NPP is hydrolysed to yellow p-nitrophenol (p-NP) in the presence of ALP and alkaline conditions, and p-NP can be quantified at an absorbance of 405 nm. The standard protocol was modified on the basis of methods described by Ji et al. [12]. Fibres and particles (10 mg, n = 3) were dissolved in deionised water (5 mL). Aliquots (80 μL) were incubated with 2-amino-2-methyl-1-propanol (1.5 M in deionised H_2_O, 20 μL; Acros Organics, UK) and loaded in a 96-well plate. The p-NPP liquid substrate solution (100 μL; Sigma Aldrich, UK) was added to the initial mixture. After 5 min, NaOH (1 M, 20 μL) was added to stop ALP catalysis and the absorbance of p-NPP was measured using a SpectraMax M2 microplate reader (Molecular Devices, USA) at 405 nm. The percentage relative ALP activity was then determined using Equation (3):

Equation 3

(3) $$ALP\;activity\;\left(\%\right)=\frac{\;ALP\;loading\;determined\;by\;ALP\;activity\;assay}{ALP\;loading\;calculated\;using\;MicroBCA™}\times 100$$

### 2.6 Statistical analysis

All data are presented as mean ± standard deviation (SD) from three independent samples unless indicated otherwise. Statistical analysis and graph plotting were performed using the OriginLab software. Statistical significance was evaluated by one-way analysis of variance (ANOVA) using Tukey's post-hoc test. Significance was set at a p-value (p) < 0.05 (*); ** denotes p < 0.01, *** denotes p < 0.001, and **** denotes p < 0.0001.

## 3 Results and discussion

### 3.1 Morphological characterisation

**Fig. 2:**
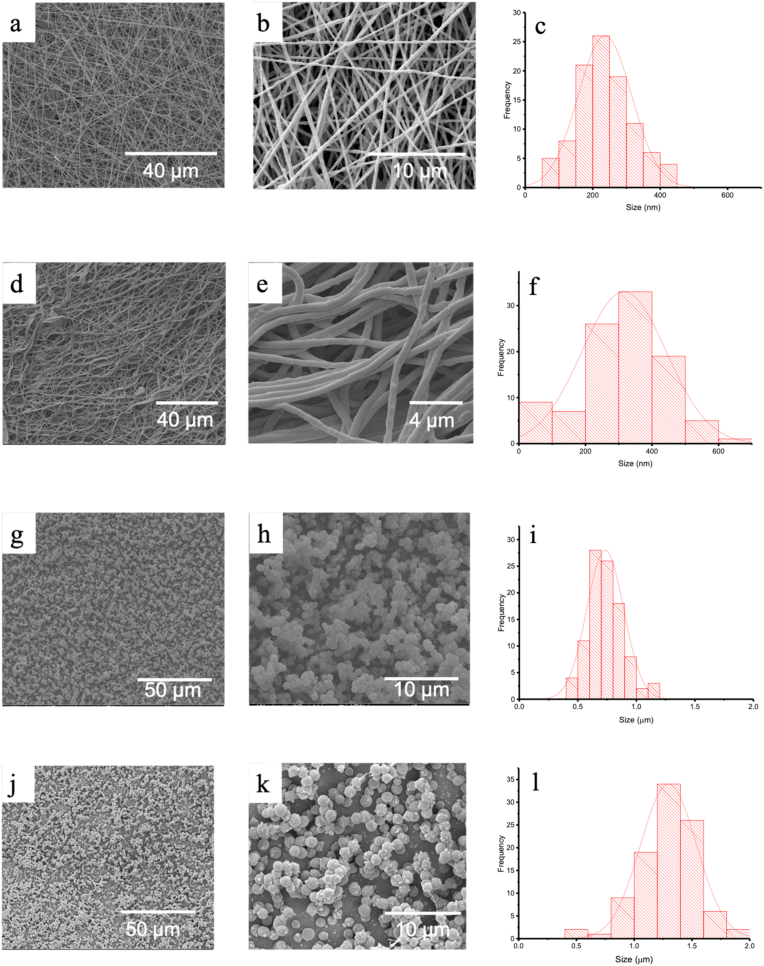
SEM images and respective size distributions (n = 100) of (a–c) EFB, (d–f) EFC, (g–i) EPB, and (j–l) EPC. The mean diameters ± SD for each formulation were 236 ± 79 nm, 316 ± 127 nm, 730 ± 160 nm, and 1290 ± 240 nm, respectively.

The morphology of electrospun formulations is affected by a number of factors such as solvent choice, material properties, the concentrations of constituent materials, and processing parameters [51]. Using the parameters in Table 2, smooth, uniform bead-free fibres (EFB) comprised of ALP and PEO could be produced by blend electrospinning, with an average diameter of 236 ± 79 nm (Fig. 2 a and b). The histogram in Fig. 2c shows the blend fibre diameters to have a normal distribution. The sizes noted here are consistent with the literature: in a study by Wongsasulak et al., blend electrospun PEO fibres containing ovalbumin had diameters ranging from 188 to 470 nm [52].

The core-shell ALP-PEO (EFC) fibres (Fig. 2d and e), prepared using the optimal conditions presented in Table 3, show bead-free cylindrical structures. These fibres have an average diameter of 316 ± 127 nm, somewhat larger than EFB and with less of a normal distribution (see Fig. 2f). In a study by Tiwari and Venkatraman, the formation of monolithic and core-shell fibres was compared [53]. The team deduced that the difference in viscosity of the solutions used in either process affects the fibres produced. For EFB, the polymer solution viscosity was reduced (cf. EFC) due to the addition of the protein solution before spinning. The ALP was dissolved in PBS, resulting in a solution much less viscous than the PEO solution, and when the two are combined for electrospinning the overall viscosity of the mixed solution will be somewhere between the two starting solutions. This reduced viscosity leads to EFB having narrower fibres than EFC.

The SEM images of the blend ALP-PEO particles (EPB; Fig. 2g and h) show monodisperse PEO particles with an average particle size of 730 ± 160 nm. The size histogram displays a normal distribution (Fig. 2i) with most particles ranging from 500 to 1000 nm in size. In turn, images of the core-shell ALP-PEO particles (EPC) in Fig. 2j and k show particles with generally smooth surfaces and an average size of 1290 ± 240 nm. There appears to be a secondary population of smaller satellite particles attached to the larger bulk (see Fig. 2k). The particle size histogram in Fig. 2l again shows a normal distribution. Comparing the blend and coaxial formulations, the EPB system appears to be more aggregated than EPC. This higher cohesion may be attributed to the presence of proteins at the particle interface in EPB, changing surface tension properties [54,55].

Both the particles and fibres that were fabricated by coaxial processes had larger diameters than their blend counterparts. Reduction in material diameter could be due to reduced PEO concentration when the protein and polymer solutions are directly blended [56]. Moreover, the presence of charged proteins at the surface of the fibres and droplets in the monoaxial experiment reduces the stability of the travelling jet, promoting breakup or fission and resulting in smaller diameters [57]. This is borne out by the literature: Reardon et al. prepared core-shell and blend PLGA microparticles using EHD processing techniques. The blend particles had an average diameter of 550 ± 80 nm, whilst the core-shell particles had an average diameter of 850 ± 200 nm [58].

**Fig. 3:**
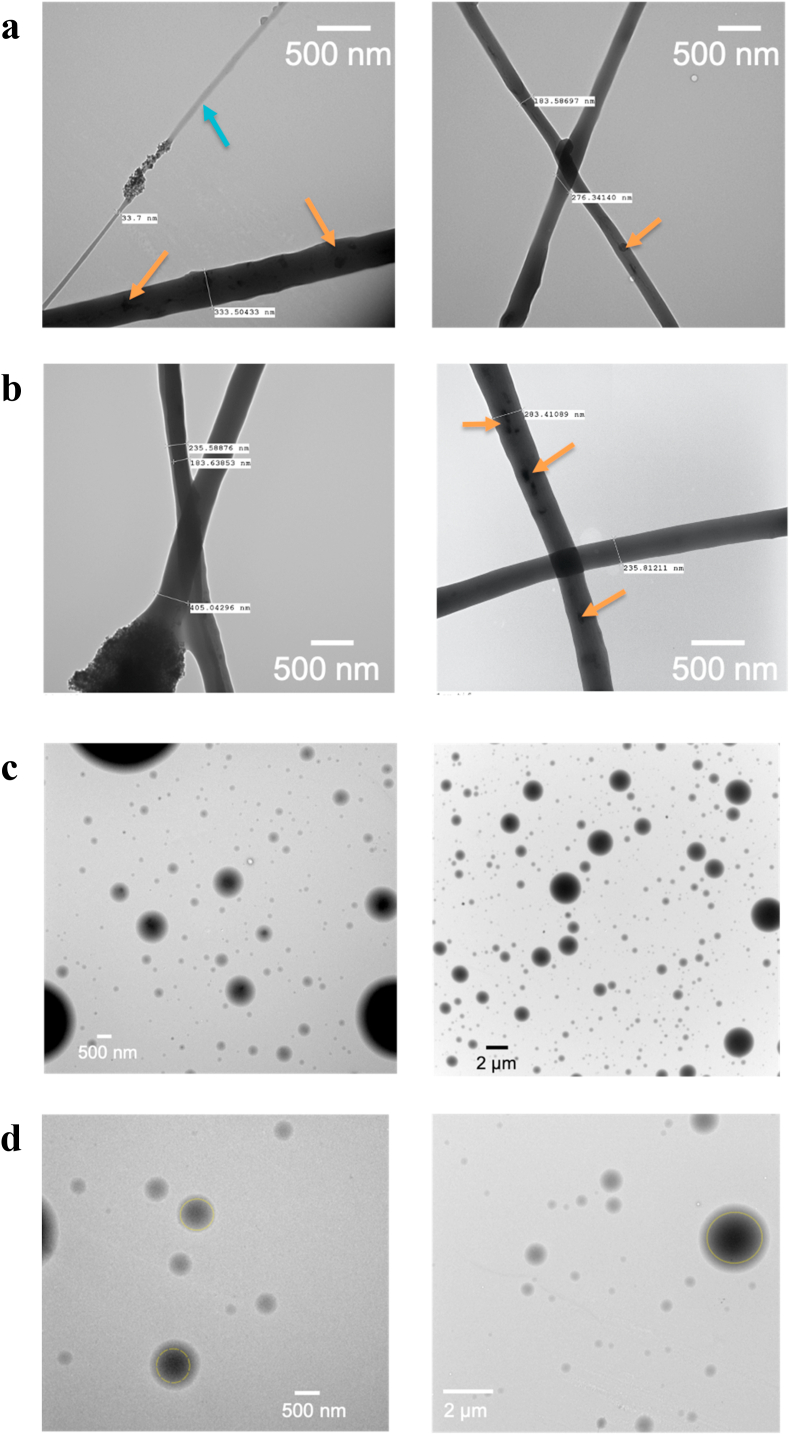
TEM images of a) EFB, with the blue arrow indicating the formation of fibres with a smaller diameter and orange arrows confirming the formation of monolithic fibres, b) EFC, with orange arrows showing the formation of core-shell structures, c) EPB, displaying homogeneous particles, and d) EPC, displaying a core-shell structure (highlighted by dashed yellow lines).

TEM images of the EFB fibres show structures varying in size (Fig. 3a). The fibre diameter measured by TEM is consistent with findings deduced from the SEM results, at ca. 300 nm. Blend fibres are known to be monolithic in nature, which holds true for the EFB fibres here [59]. In contrast, the TEM images in Fig. 3b show a core-shell structure for the EFC sample, with an internal diameter of approximately 185 nm and a shell diameter of 235 nm. The EFC diameter of 235 nm falls within the size distribution expected for this sample from SEM (as detailed in Fig. 2f). The fact that the TEM diameter is somewhat smaller than the mean SEM value can be ascribed to the low number of observations in the TEM experiment. In the TEM images of both EFB and EFC there seem to be some aggregates present in the fibres, likely to correspond to ALP agglomeration.

TEM images of the electrosprayed particles are shown in Fig. 3c (EPB) and Fig. 3d (EPC). The images for EPB suggest the formation of homogenous particles. The TEM images of the coaxial particles are consistent with core-shell structures having been formed, presumably with ALP being localised within the particle core and enveloped by the PEO shell.

These findings are all consistent with previous studies by other groups. Wen et al. utilised coaxial electrospinning for the encapsulation of a BSA/chitosan core in a sodium alginate and PEO shell, and achieved distinct core-shell structures [60]. Other examples of core-shell particles using coaxial EHD processes have been described, including for stem cell encapsulation in collagen [61] and BSA encapsulation in PLGA [62].

### 3.2 Physicochemical characterisation

**Fig. 4:**
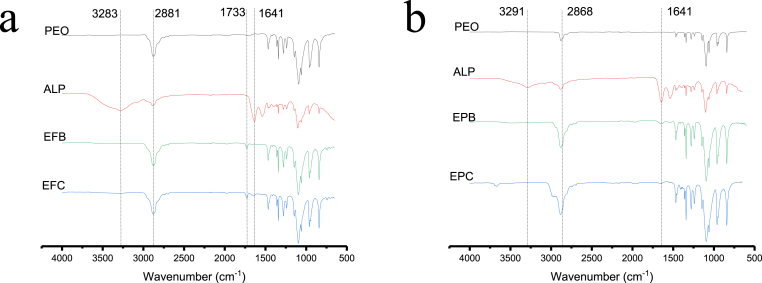
FTIR spectra of a) as supplied PEO (600 kDa), ALP, EFB and EFC. b) FTIR spectra of as supplied PEO (20 kDa), ALP, EPB and EPC. The spectra show ALP bands are present in both the fibres and particles.

FTIR spectra are displayed in Fig. 4. The PEO absorption peaks at around 1341 cm^−1^ are assigned to vibrations of the C-H bonds. Bands at around 1064 cm^−1^ and ca. 1100 cm^−1^ are C-O-C and C-C group stretching vibrations. The peak at 1460 cm^−1^ arises from C-H bending [22]. The main peaks identified in ALP include a broad peak at 3283 cm^−1^ that corresponds to the presence of a secondary amine (N-H). The primary amine band is located at 1641 cm^−1^ (amide I) and is closely followed by amide II, an NH_2_ bending peak at around 1530 cm^−1^. Both these peaks are present – although weak – in all the ALP loaded fibres and particles, confirming protein encapsulation. The reduced intensity of the ALP peaks in the formulations is presumably owing to the protein comprising a relatively small proportion of the overall mass of the material [63].

All the key bands of PEO are observed in the spectra of the formulations generated by EHD processes. The absorption peak at 2881 cm^−1^ for the fibres and 2868 cm^−1^ for the particles is attributed to CH_2_ bending vibrations in both the polymer and protein [22]. In the spectra for the blend and core-shell fibres, a weak absorption peak is identified at 1733 cm^−1^. This peak is not present in the polymer or protein and is indicative of a carbonyl stretch. This carbonyl stretch could arise due to a shift in the original amide band position, or it may have appeared as a result of hydrolysis or oxidation of the protein due to the complexity of the structures being processed [63].

DSC and XRD data were also collected on the formulations (Supplementary Information, Figs. S1 and S2). These are dominated by the features of PEO, as would be expected given that it comprises the bulk of the mass of the particles and fibres. All the formulations contain ALP amorphously distributed in a semi-crystalline PEO matrix.

### 3.3 Encapsulation efficiency and drug loading

The ALP loading of both blend and coaxial fibres was determined using MicroBCA™ assays (Table 6).

**Table 6:** Drug loading and encapsulation efficiency electrospun fibres and electrosprayed particles loaded with ALP (data presented as mean ± S.D., n = 3).

	Theoretical loading	Drug loading	Encapsulation efficiency
EFB	19.2%	16.6 ± 0.6% (w/w)	86.2 ± 2.9%
EFC	21.7%	20.6 ± 1.2% (w/w)	94.6 ± 5.4%
EPB	5.9%	5.0 ± 0.2% (w/w)	85.0 ± 4.0%
EPC	3.0%	3.0 ± 0.4% (w/w)	99.0 ± 12.0%

Table 6 reveals that blend fibres contain less ALP than the core-shell fibres. The encapsulation efficiency is also lower for EFB, which could be due to the mechanical dispersion method used for the blend structure: gentle mixing was used, which may not have resulted in a fully homogeneous solution (homogeneity of protein distribution in a polymer solution can be hard to achieve without the use of high shear equipment) [64]. In addition, the direct exposure of the protein to organic solvent in the blend formulation could have resulted in the precipitation of some enzyme [65].

Chew and colleagues investigated the encapsulation of human β-nerve growth factor (NGF) and BSA in electrospun monolithic fibres. They calculated theoretical loadings of 0.0123 and 4.08% for NGF and BSA respectively [57], but observed significantly lower levels of encapsulated NGF (3.10 × 10^−4^%), attributing this difference to instability of the protein-polymer jet during electrospinning. BSA levels were not determined. The evidence from Chew's study suggests that charged materials, such as proteins, could act very differently from the polymer carrier in an electromagnetic field, causing them to be deposited on surfaces that might not have been intended for collection. The low loading efficiency for protein actives can be improved by processing separate solutions of the protein and polymer solutions in coaxial electrospinning [66]. Protein encapsulation efficiency is generally increased in core-shell structures as the core solution is within the shell and carried with it to the collector, so drying typically occurs before the protein can migrate to the surface of the material. The results from Chew's study corroborate the findings in Table 6, as encapsulation efficiency was improved in core-shell structures [57].

As observed for the fibre preparations, the encapsulation efficiency of EPB was lower than that of EPC (Table 6). However, the ALP loading was greater for EPB than EPC, owing to the low flow rate which had to be used for the core of EPC. When comparing the drug loading of fibres to that of particles, more ALP is loaded in the former. Differences in drug loading arise due to the concentration of protein in the original stock solution and the ratio of polymer to protein in the feedstock, which affects protein concentration as described in Sections 2.1, 2.2, and to the flow rate of the protein feed [67]. For example, the shell solution for fibre formation was flowing 6 times faster than the core protein solution, whilst for the particles the shell flowed 15 times faster. It was found to be easier to form fibres than particles and optimisation of particle production required significant reduction of the core flow rate, which resulted in the reduced protein loading in the latter (Table 6).

### 3.4 SDS-PAGE

**Fig. 5:**
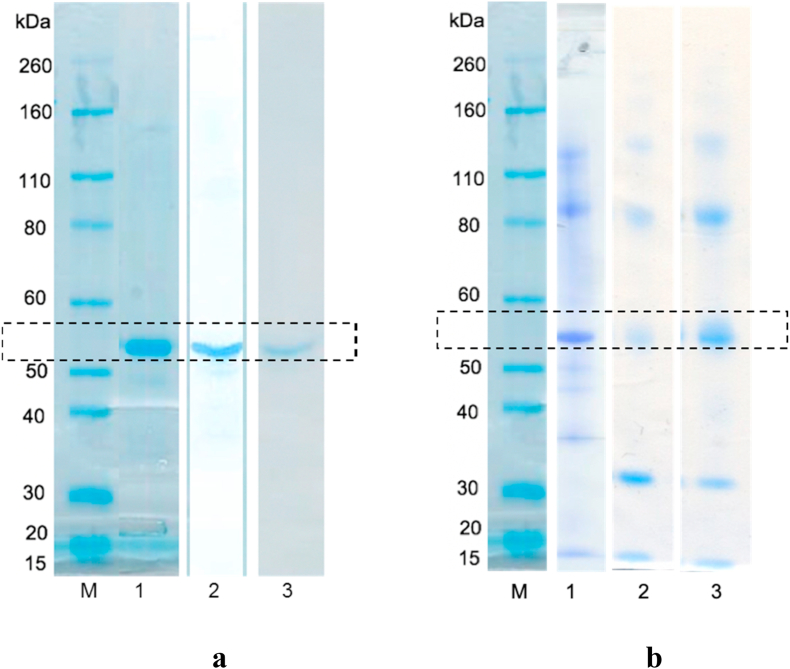
SDS-PAGE gels for ALP after encapsulation into a) particles and b) fibres. M denotes the marker lane. Bands in a) correspond to: 1) ALP, 2) EPB and 3) EPC. Bands in b) represent: 1) ALP, 2) EFB and 3) EFC.

Experiments were carried out to determine the effect of each EHD process on ALP. The gels show clear protein bands at around 56 kDa from ALP, both for fresh ALP and after dissolution of EPB and EPC particles (Fig. 5a). As ALP is expected to exist as dimers with a molecular weight of 115–165 kDa, the position of the bands suggests the as-supplied ALP was broken down into monomers [68], either during the freeze-drying process used by the manufacturer or by the SDS used in the gel. Nevertheless, this suggests that the preparation of ALP-loaded blend and core-shell particles did not cause any fragmentation or aggregation of the protein.

For the fibres (Fig. 5b), the SDS-PAGE data are rather different: freshly dissolved ALP shows bands at 100–120 kDa (dimers), as well as around 55 kDa (monomers). This can be explained by the fact that different batches of ALP were used for fibre and particle fabrication. After spinning, these bands are all still present, but a new band appears with a size of around 30 kDa. This might be the result of breakdown products which may also be linked to the additional FTIR C=O band at 1733 cm^−1^ noted after electrospinning (Fig. 4). Nonetheless, both blend and coaxial electrospinning and electrospraying appear to generally maintain the integrity of ALP after processing, and there are no clear differences noted between coaxial and monoaxial methods.

To check whether the differences in unprocessed ALP observed in the two gels in Fig. 5 could be attributed to the distinct protein batches used for particles and fibres, this experiment was repeated with a third protein batch, and the corresponding gel is in Fig. S3 (Supplementary Information). This third band profile is different from both those presented in Fig. 5: an extra band of higher molecular weight (close to 260 kDa) and three individual bands at around 35 kDa can be observed in this gel, suggesting that the as-supplied ALP may have suffered some structural modifications, such as aggregation and hydrolysis. This variation in the composition of ALP batches is consistent with the DSC data (Fig. S1). Nevertheless, it is clear that ALP released from EFB and EFC had similar bands to those of the freshly dissolved enzyme, therefore confirming that EHD processing has no visibly nefarious effects in protein integrity.

### 3.5 ALP activity

**Fig. 6:**
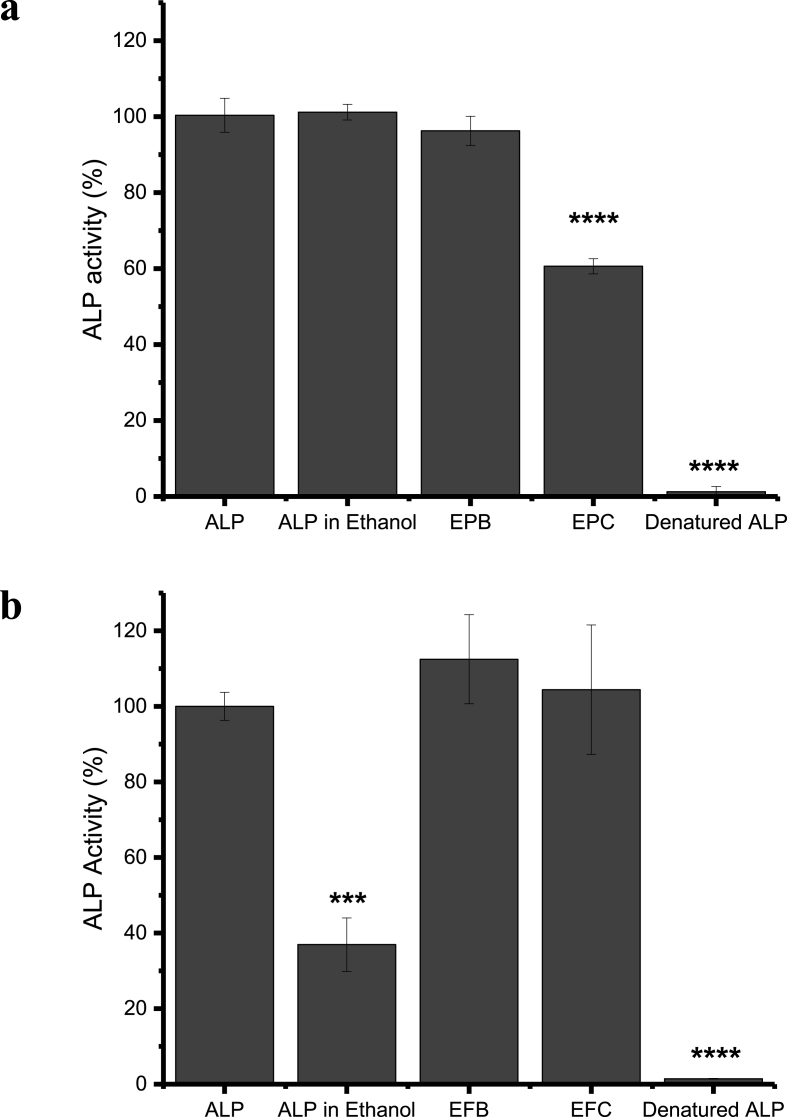
The results of ALP activity assays for a) electrosprayed particles and b) electrospun fibres. Data are reported as mean ± S.D. (n = 3). The majority of ALP remained active after fabrication except in the case of EPC. Fresh ALP was used as a positive control and ALP boiled in water at 100 °C for an hour (denatured ALP) was the negative control. *** denotes p < 0.001, **** denotes p < 0.0001.

ALP activity assays were employed to assess whether there was any loss of activity caused by EHD processing. Activity was calculated relative to the ALP concentration, previously determined from the MicroBCA™ assay. The results in Fig. 6a indicate that the ALP in EPB retained almost all its activity, whilst that in EPC retained approximately 60%, with a statistically significant difference (p < 0.0001**)** in protein activity when compared to fresh ALP. Conversely, in Fig. 6b, it is possible to see that both blend and core-shell electrospun fibres retained approximately 100% of the ALP activity.

In an attempt to obtain more understanding as to which facet of the EHD processing might have led to loss in activity (organic solvent exposure or application of electrical field), a control experiment was performed with ALP dissolved directly in ethanol, the solvent employed for EHD. There is a marked disparity between the enzymatic activity of fresh ALP dissolved in ethanol in the two experiments, which is presumably a consequence of the different batches used (Section 3.4). What is more, it was possible to dissolve the batch of ALP used for particle production (Fig. 5a) directly in ethanol, while the batch used for fibre fabrication (Supplementary Fig. S3) had to be pre-dissolved in a small volume of deionised water before diluting in absolute ethanol. This further demonstrates that the inherent fluctuation in structural integrity associated with as-supplied ALP results in varying physical properties. After extraction from EFB and EFC the activity of ALP is identical to or higher than that of ALP freshly dissolved in ethanol, therefore confirming that the presence of ethanol in the polymer solutions did not compromise protein integrity.

It is thus not expected that the loss of ALP activity in the EPC formulation (Fig. 6a) was due to contact of the enzyme with the ethanol present in the shell fluid: this also occurred during the production of EFC and, to an even greater extent, during blend electrospinning (EFB) and spraying (EPB). It is thus hypothesised that the decrease in ALP activity in core-shell particles may be attributed to the high voltage (22.5 kV) required for processing. In the literature, Krishnaswamy and Kenkare investigated the effect of organic solvents such as dioxane (25% v/v in water) and formamide (25% v/v in water) on ALP activity [69]. ALP appears to maintain activity when exposed to mixed aqueous-organic solvent systems. While there may have been some protein denaturation, the ability of the enzyme to transform the p-NPP substrate was still maintained [69]. This may also be the case for ALP in the presence of binary mixtures of ethanol and water, explaining the retention of activity for EPB, EFB and EFC. The results presented here, together with those from Krishnaswamy's study, suggest that core-shell processes are not always necessary, and protein stability in the solvent system of interest should first be investigated prior to determining the most appropriate EHD technique to be used.

In contrast, Tiwari and Venkatraman investigated the effect of organic solvents (chloroform and dimethylformamide mixtures) on lysozyme encapsulated in blend PLGA fibres fabricated by electrospinning [70]. It was found that increasing concentrations of dimethylformamide caused a 30% reduction in lysozyme activity, presumably due to loss of the enzyme's tertiary structure [70]. The presence of the organic solvents in addition to a strong electric field (22–25 kV) further reduced lysozyme enzymatic function, until only 36% of activity was retained after electrospinning. Conversely, Kim et al. electrospun fibres of lysozyme in PCL and PEO using a monoaxial approach at a voltage of 15 kV and a chloroform and DMSO solvent blend, but they only found a reduction in lysozyme activity of about 5–10% [11].

Advances in food technology have resulted in the use of pulsed electric fields (PEF) as a non-thermal method to minimise bacterial growth but preserve the nutritional value of liquid and semi-liquid foods [71]. Shamsi et al. investigated the effect of PEF on ALP inactivation. The group discovered that PEF treatments of 25–35 kV at 15 °C caused a decrease of 24–42% in ALP catalytic activity [72]. Another study found that pulses of 22.3 kV reduced ALP activity by 44% [73]. These studies indicate that there may be changes in ALP activity due to exposure to high voltage. The EPC was prepared at the highest voltage of 22.5 kV and also used a relatively slow flow rate, resulting in an increased exposure time of ALP to the electric field. The remaining formulations were processed using notably lower voltages of 10–15.5 kV. The loss of ALP activity observed for EPC but not for EFC may thus be primarily due to prolonged exposure to a high voltage in the former case. Coaxially processed systems, especially particles, typically require higher voltage than blend EHD systems. Hence, although this work shows that higher encapsulation efficiency can be achieved using core-shell processes, care has to be taken during EHD processing so as not to cause electric field-induced protein inactivation.

## 4 Conclusions

ALP-PEO fibres and particles were fabricated by monoaxial and coaxial EHD processes. The former led to monolithic products and the latter to core/shell systems, as would be expected. All the formulations comprised amorphously distributed ALP in a semi-crystalline PEO carrier. The encapsulation efficiencies were lower for the blend formulations than the core-shell analogues, because directly mixing the protein and polymer can cause the jet to deposit in areas other than the collector during electrospinning and spraying. Electrospinning resulted in higher ALP loading than electrospraying, but no major differences were found between the encapsulation efficiencies of fibres and particles. The results from the activity assays reveal that the EHD processes used to prepare both particles and fibres maintained ALP activity, except in cases where the solution being processed was exposed to very high voltages (in the case of the core/shell EPC particles from coaxial electrospraying). After EHD processing, the tertiary structure of ALP appeared unchanged in SDS-PAGE investigations, but its activity was reduced by about 40% when coaxial electrospraying was used to prepare particles.

This work lays a foundation for the investigation of protein and peptide delivery systems, and for the consideration of an optimal EHD technique to be employed in a given setting. It appears that exposure of a protein to some organic solvent during blend processing does not necessarily impair activity and structural integrity, and thus coaxial methods are not always needed. In the case of ALP, blend techniques were shown to be as or more effective at preserving protein activity than coaxial EHD processing, offering an opportunity to explore less complex means of protein formulation.

## Credit statement

LCO: conceptualisation, formal analysis, investigation, methodology, validation, visualisation, writing – original draft, writing – review & editing. AM: conceptualisation, formal analysis, investigation, methodology, validation, visualisation, writing – original draft, writing – review & editing. JZ – investigation, methodology, writing – review and editing. UA – conceptualisation, methodology, validation, writing – review & editing PFC – supervision, funding acquisition, project administration, writing – review and editing. SB - conceptualisation, formal analysis, funding acquisition, project administration, supervision, writing – review & editing. GRW – conceptualisation, formal analysis, funding acquisition, project administration, supervision, writing – review & editing.

## Declaration of competing interest

The authors confirm that they have no conflicts of interest.
