# Supplementary material for: An investigation of alkaline phosphatase enzymatic activity after electrospinning and electrospraying
Source: J Drug Deliv Sci Technol. 2021 Aug;64:None. doi: 10.1016/j.jddst.2021.102592 (PMC8312041; doi:10.1016/j.jddst.2021.102592)
Supplement: Multimedia component 1 [file mmc1.docx]

# Supplementary Information for

# An investigation of alkaline phosphatase enzymatic activity after electrospinning and electrospraying

Lesley C. Onyekuru^1^, Anabela Moreira^2^, Jiazhe Zhang^1^, Ukrit Angkawinitwong,^1^ Pedro F. Costa^2^, Steve Brocchini^1,3^, Gareth R. Williams^1^

^1^ UCL School of Pharmacy, University College London, 29-39 Brunswick Square, London WC1N 1AX, UK

^2^ Biofabics Lda., Rua Alfredo Allen 455, 4200-135 Porto, Portugal

^3^ UCL Institute of Ophthalmology, University College London, 11-43 Bath Street, London EC1V 9EL, UK

| a |  | b |  |
| --- | --- | --- | --- |

Figure S1. DSC thermograms of a) as supplied PEO (600 kDa), electrospun (ES) PEO, ALP, EFB and EFC; b) as supplied PEO (20 kDa), ALP, EPB and EPC. The thermograms show EHD processed materials closely maintained the proprieties of their constituent PEO polymers. Sharp endotherms corresponding to the melting temperature (T_m_) of PEO (ca. 66 °C) are seen in all formulations. The melting temperature of the fibres is generally higher than the particles owing to the difference in the molecular weights of the PEO used (20 kDa for the particles and 600 kDa for the fibres) [1]. For the fibres, the melting enthalpy of the PEO-containing materials reduced from 28.6 J/g to ca. 23 J/g for both EFB and EFC, suggesting a reduction in crystallinity for the 600 kDa polymer post-processing. In the particles, the melting enthalpy of PEO (20 kDa) decreased from 30.7 J/g to 23.9 J/g and 17.3 J/g for EPB and EPC, respectively. In the particle formulations, there is a greater reduction in the melting enthalpy of the core-shell processed material than that of the blend, suggesting that core-shell processing of particles increases the amorphous regions of the polymer. ALP alone displays an endotherm below 60 °C, which corresponds to the degradation of the enzyme [2]. The batch of ALP employed to prepare the fibres (a) has a different degradation temperature than that of the ALP in the particles (b), suggesting a difference in the physical properties of the protein used.

| a |  | b |  |
| --- | --- | --- | --- |

Figure S2. XRD patterns of a) as supplied PEO 600 kDa, ALP, EFB, and EFC; b) as supplied PEO 20 kDa, ALP, EPB, and EPC. The XRD pattern for ALP shows a halo in both a) and b), with no Bragg reflections, due to the amorphous nature of the as-supplied freeze-dried ALP [3]. PEO-containing samples are observed to have Bragg reflections at around 19°, 24° and 27°, which are characteristic of semi-crystalline PEO [4]. There are some difference in the patterns of the formulations compared to that of blank PEO, indicating changes in the crystal packing post-EHD processing. The higher molecular weight PEO does not have a peak at 27° after fibre fabrication, but a detailed analysis of the pattern reveals a much weaker Bragg reflection at around 32°. These data, combined with the DSC results (Figure S1), suggest that both particles (EPB and EPC) and fibres (EFB and EFC) contain ALP amorphously distributed in a semi-crystalline PEO matrix.


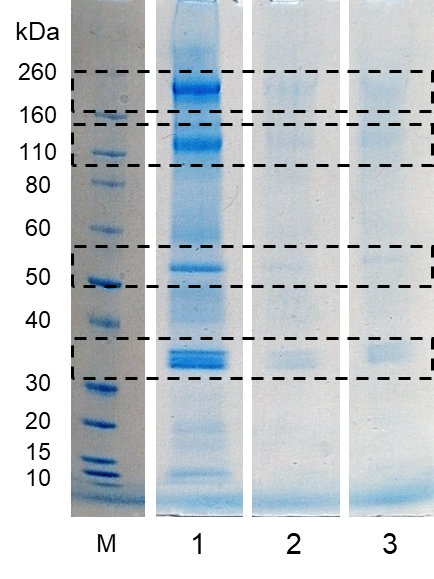


Figure S3. SDS-PAGE gel of ALP released from EFB and EFC, with the formulations prepared from a third distinct protein batch. Lanes: M) marker, 1) ALP, 2) EFB and 3) EFC. It is possible to observe that this profile differs from those presented in Figure 5: even though no differences between fresh and encapsulated ALP can be found, it is clear that unprocessed ALP already presents some structural alterations, given the multiple high and low molecular weight bands observed (potentially indicating protein aggregation and hydrolysis, respectively).

**References**

[1] K. Pielichowski, K. Flejtuch, Phase Behavior of Poly(Ethylene Oxide) Studied by Modulated-Temperature DSC - Influence of the Molecular Weight, J. Macromol. Sci. - Phys. 43 B (2004) 459–470. https://doi.org/10.1081/MB-120029781.

[2] L.F. Atyaksheva, B.N. Tarasevich, E.S. Chukhrai, O.M. Poltorak, Thermal inactivation of alkali phosphatases under various conditions, Russ. J. Phys. Chem. A. 83 (2009) 318–323. https://doi.org/10.1134/S0036024409020307.

[3] L.L. Chang, M.J. Pikal, Mechanisms of protein stabilization in the solid state, J. Pharm. Sci. 98 (2009) 2886–2908. https://doi.org/10.1002/jps.21825.

[4] A.Y.A. Kaassis, N. Young, N. Sano, H.A. Merchant, D.-G. Yu, N.P. Chatterton, G.R. Williams, Pulsatile drug release from electrospun poly(ethylene oxide)–sodium alginate blend nanofibres, J. Mater. Chem. B. 2 (2014) 1400–1407. https://doi.org/10.1039/C3TB21605E.
